# Impacted large ureteral stone: What is the best approach?

**DOI:** 10.1590/S1677-5538.IBJU.2019.0638.1

**Published:** 2020-11-18

**Authors:** Fábio C. M. Torricelli

**Affiliations:** 1 Universidade de São Paulo Faculdade de Medicina da Divisão de Urologia do Hospital das Clínicas São PauloSP Brasil Divisão de Urologia do Hospital das Clínicas da Faculdade de Medicina da Universidade de São Paulo. São Paulo, SP, Brasil

## COMMENT

Treatment of impacted large ureteral stone is a challenging procedure for endourologists. Several options are available including retrograde ureteroscopy (URS), anterograde percutaneous access, shockwave lithotripsy (SWL) and transperitoneal or retroperitoneal laparoscopic ureterolithotomy (1–3). EAU and AUA guidelines recommend retrograde flexible ureteroscopy or percutaneous approach as first options for large ureteral stones management based on their high stone-free rate and minimal invasiveness (4, 5). Gökce et al. performed an interesting study comparing anterograde and retrograde access for large ureteral stone in elderly population (6). They have reported a higher stone-free rate with mini-percutaneous access (16 Fr) and similar complication rate when compared to URS. Main limitations were the lack of randomization process and no sample size calculation in the methodology. Another point the deserve attention was the low frequency of flexible device in the percutaneous access (7%, 5 of the 68 cases).

Previous studies have demonstrated the elderly population when submitted to percutaneous nephrolitotomy (PCNL) can experience more complications and longer hospital stay (7, 8). In a systematic review and meta-analysys, De et al. have reported that PCNL is associated with higher stone-free rate at the expense of higher complication rate, blood loss, and admission time when compared to retrograde intrarenal surgery (9). Mini-percutaneous access seems to be an option to minimize surgical complications, especially in high-risk patients as elderly. Gao et al. have reported the outcomes of a systematic review and meta-analysis including 5 randomized clinical trials comparing mini-PCNL and URS for the treatment of large ureteral stones. Mini-percutaneous access provided higher stone-free rate and similar complication rate than URS. URS had a shorter hospital stay (10).

In a recent published systematic review and meta-analysis including 12 randomized clinical trials and 1416 patients comparing laparoscopic ureterolithotomy (LU), PCNL and URS, authors have found that PCNL and LU achieved a higher stone-free rate and a lower ureteral injury rate than URS (1). In another systematic review and meta-analysis including 25 studies and 2888 patients comparing SWL, PCNL, URS and LU for large ureteral stone management, authors have reported LU as the method with higher stone-free rate and complication rate only superior to SWL (3). These meta-analyses show that endourolgists who have experience with laparoscopic surgery have one more interesting option when deciding the best approach for an impacted large ureteral stone.
